# 1-Methyl-D-Tryptophan Potentiates TGF-β-Induced Epithelial-Mesenchymal Transition in T24 Human Bladder Cancer Cells

**DOI:** 10.1371/journal.pone.0134858

**Published:** 2015-08-12

**Authors:** Rodrigo Barbosa Oliveira Brito, Camila Soares Malta, Diego Mota Souza, Luiz Henrique Gomes Matheus, Yves Silva Teles Matos, Chrisna Souza Silva, Janaína Mendes Ferreira, Valeria Sutti Nunes, Cristiane Miranda França, Humberto Dellê

**Affiliations:** 1 Programa de Pós-graduação em Medicina, Universidade Nove de Julho (UNINOVE), São Paulo, Brazil; 2 Lipids Laboratory (LIM-10), Endocrinology and Metabolism Division of Hospital das Clínicas, Faculty of Medical Sciences, University of São Paulo, São Paulo, Brazil; 3 Programa de Pós-graduação em Biofotônica em Ciências da Saúde, Universidade Nove de Julho (UNINOVE), São Paulo, Brazil; University Hospital of Heidelberg, GERMANY

## Abstract

Immune escape and metastasis are the hallmarks of several types of cancer including bladder cancer. One of the mechanisms involved in these processes has been linked to indoleamine 2,3-dioxygenase (IDO). Although IDO is classically recognized for its immunomodulatory property, it has presented nonimmunological effects in some tumors. TGF-β1 is believed to contribute to carcinoma development by modulating immunossupressive molecules, including IDO. In addition, TGF-β1 induces the epithelial-mesenchymal transition (EMT), which is a critical step in the tumor invasiveness and metastasis. We investigated the role of MT and IDO modulation in the induction of EMT by TGF-β1 in T24 human bladder carcinoma cells. When T24 cells were incubated with the IDO inhibitor (MT, 1-methyl-D-tryptophan), with TGF-β1, and with MT+TGF-β1, a significant decrease of IDO expression and activity was observed. In addition, downregulation of e-cadherin and upregulation of n-cadherin and EMT transcription factors were induced by the treatments, confirming the induction of EMT. siRNA-mediated knockdown of IDO decreased e-cadherin expression, but had no effect on EMT transcription factors. In the scratch-wound assay, the heightened migration process was intensified when the cells were incubated with MT+TGF-β1. These effects were associated with a robust inhibition of Akt activation. After inoculation of T24 cells under the kidney capsule of Balb/c nude, the cells were positive for IDO in the center of the cell infiltrate, being negative in the periphery, where EMT is high. In conclusion, inhibition of IDO by TGF-β1 and MT is associated with EMT in T24 human bladder carcinoma cells. MT has potentiating effect in TGF-β1-induced EMT, independently of IDO. This nonimmunological effect of MT should be considered if IDO is the target to avoid immune escape in bladder cancer.

## Introduction

Urinary bladder cancer is the most common malignancy of the urinary system [1]. Although the most common form of human bladder cancer is non-muscle invasive (70% to 80%), 30% to 50% of these cases progress to a muscle-invasive form after repeated resections, which ultimately leads to cancer-specific death and metastasis [2].

Multiple mechanisms have been implicated in tumor invasiveness and metastasis, including the epithelial-mesenchymal transition (EMT). In this process, a polarized epithelial cell assumes a mesenchymal phenotype, which leads to a loss of cellular adhesion to the basement membrane, activation of motility and invasiveness, and production of extracellular-matrix-degrading enzymes [3]. Several molecules are engaged in the initiation of EMT, including the TGF-β superfamily proteins. TGF-β1 is an important inducer of EMT in several different types of tumors [4], including bladder cancer [5]. Certain genetic variations and increased plasma levels of TGF-β are potent predictors of bladder cancer risk [6,7].

Indoleamine 2,3-dioxygenase (IDO) is an enzyme that catalyzes the degradation of the amino acid tryptophan, which leads to accumulation of tryptophan metabolites, such as kynurenine. As described by Munn et al., IDO has a role in the maternal-fetal interface and functions to protect embryos against the maternal immune system [8]. Subsequently, IDO has been explored for use as an effective immunomodulatory molecule. Because IDO is produced by many cell types of the immune system, including dendritic cells, macrophages, and regulatory T cells, IDO has been implicated in immune-mediated disorders, such as allograft rejection [9], autoimmune diseases [10] and escape from antitumor immunity in cancer [11]. Regarding cancer, IDO activity is present in many human tumor types as well as tumor-draining lymph nodes [11]; however, the role of IDO in tumor growth is still poorly understood. While IDO expression is correlated with a less favorable prognosis for many types of cancer including colorectal [12], uterine cervical [13], endometrial [14], breast [15], melanoma [16], ovarian [17], and lung cancer [18], IDO expression is also associated with recurrence-free survival of hepatocellular patients [19] and the long-term survival of patients with renal carcinoma [20]. Although there is a controversy about the role of the IDO in cancer, molecules that can modulate IDO-mediated pathways have been seen as promising for cancer treatment. In this context, 1-methyl-D-tryptophan, a competitive inhibitor of IDO, has been intensively studied as anticancer agent. Currently, the association of 1-methyl-D-tryptophan (MT) with docetaxel was used in a phase I clinical trial with patients with metastatic solid tumors [21]. However, this molecule may act independently of IDO activity [22], as well as like tryptophan to regulate mTOR pathway [23].

IDO expression has been found not only in tumor-infiltrating immune cells and tumor-draining lymph nodes but also in neoplastic cells. In bladder cancer, the T24 human transitional carcinoma cell line produces IDO constitutively in culture [24], even without involvement of the immune system, leading to the hypothesis that IDO may have a role in non-immune processes. Levina et al., demonstrated that upregulation of IDO in breast cancer cells increased cell proliferation and decreased apoptosis in a manner that was independent of the immunological effects of IDO [25].

Interestingly, TGF-β induces a tolerogenic phenotype in immunogenic dendritic cells, and this effect is mediated by IDO through activation of the PI3K/Akt pathway [26]. Because TGF-β induces the EMT in T24 bladder carcinoma cells [27, 28], we hypothesized that modulation of IDO may be associated with TGF-β in the induction of EMT in T24 carcinoma cells. In this study, we analyzed the effect of MT and the siRNA-mediated knockdown of IDO in the TGF-β-induced EMT in T24 bladder carcinoma cells.

## Materials and Methods

### Cell culture

Human bladder cancer T24 cells (HTB-4; American Type Culture Collection-ATCC, Manassas, VA, USA) were acquired from the cell bank of the Federal University of Rio de Janeiro. T24 cells were cultured in McCoy’s 5A Medium (Sigma-Aldrich, St. Louis, MO) supplemented with 10% fetal bovine serum and penicillin-streptomycin (Sigma-Aldrich, St. Louis, MO) and maintained at 37°C with 5% CO_2_.

To analyze the effect of TGF-β1 on IDO expression, T24 cells were seeded in 6-well plates (1X10^5^ cells per well). The cells were incubated with 1 ng/ml, 5 ng/ml or 10 ng/ml of TGF-β1 (R&D Systems Inc., Minneapolis, MN) in serum-free McCoy’s 5A Medium for 48 h (triplicate).

After determining the optimal concentration of TGF-β1 (5 ng/ml), T24 cells were seeded in 6-well plates and cultured until reaching 75% confluence. The cells were then maintained in serum-free McCoy’s 5A Medium for 48 h under the following four conditions in triplicate: only medium (control); medium containing 1 mM methyl-tryptophan (MT; 1-methyl-D-tryptophan, cat 452483, Sigma-Aldrich, St. Louis, MO); medium containing TGF-β1 (5 ng/ml); and medium containing MT plus TGF-β1. At the end of 48 h, supernatants were collected for kynurenine measurement, and the cellular monolayers were trypsinized for RNA extraction. This experiment was repeated for protein isolation.

### Small interfering RNA (siRNA) transfection

In order to knockdown endogenous IDO, T24 cells were transfected with siRNA for IDO (Silencer Select inventoried 4392420, Ambion, Carlsbad, CA) or with a non-targeting control siRNA for transfection control (Silencer Select Negative Control inventoried 4390843, Ambion, Carlsbad, CA), using Lipofectamine 3000 Reagent (Invitrogen, Carlsbad, CA). Briefly, the cells were cultured in 6 well-plates, and then were maintained in serum-free McCoy medium containing (per well) 1 μg of siRNA, 5 μl of P3000 Reagent, and 5 μL of Lipofectamine 3000 Reagent, during 24 h. After transfection, the medium was removed and the cells were cultured for 24 h in McCoy’s 5A Medium supplemented with 10% fetal bovine serum at 37°C with 5% CO_2_. After this recovery time, the cells were incubated with TGF-β1 (5 ng/ml, for 48 h, in serum-free McCoy medium), exactly as previously described. The following four conditions in triplicate were performed: T24 cells previously transfected with control siRNA (si-Control), without TGF-β 1; T24 cells previously transfected with IDO siRNA incubated without TGF-β 1 (siIDO); T24 cells previously transfected with control siRNA incubated with TGF-β 1 (TGF-β); and T24 cells previously transfected with IDO siRNA incubated with TGF-β1 (siIDO+TGF-β).

### RT-qPCR

Total RNA was extracted using PureLink RNA Mini Kit (Ambion Inc, Carlsbad, CA), according to the manufacturer’s instructions, and the first strand cDNA was synthesized by M-MLV (Promega, Madison, WI) after DNase treatment (Promega, Madison, WI). Real-time PCR was performed using Power SYBR Green PCR Master Mix (Applied Biosystems, CA). Specific primers for TBP (*TATA box binding protein*; forward 5’-TTCGGAGAGTTCTGGGATTGTA–3’ and reverse 5’–TGGACTGTTCTTCACTCTTGGC–3’; accession number NM003194), IDO (forward 5’-GGTCATGGAGATGTCCGTAA–3’ and reverse 5’–ACCAATAGAGAGACCAGGAAGAA-3’; accession number NM002164), E-cad (forward 5’-GGTCATGGAGATGTCCGTAA–3’ and reverse 5’–ACCAATAGAGAGACCAGGAAGAA-3’; accession number Z18923), N-cad (forward 5’–TGCCCGGTTTCATTTAGGGG–3’ and reverse 5’–TCCCTCAGGAACTGTCCCAT–3’; accession number X57548), TWIST (forward 5’–AGAGATGCAACTAAGCCCTCT–3’ and reverse 5’–AAGCAGCTACTGACAGGCAC–3’; accession number Y11177), Snail (forward 5’–ACCACTATGCCGCGCTCTT–3’ and reverse 5’–GGTCGTAGGGCTGCTGGAA–3’; accession number NM005985), and Slug (forward 5’–TGTTGCAGTGAGGGCAAGAA–3’ and reverse 5’–GACCCTGGTTGCTTCAAGGA–3’; accession number NM003068) were used. Triplicate of each sample were heated at 95°C for 5 min and then subjected to 40 cycles of denaturation at 95°C for 15 sec, annealing at 60°C for 60 sec and extension at 60°C for 60 sec. These reactions were performed using an Applied Biosystems 7500 Real-Time PCR System (Applied Biosystems, Ca, USA). After real-time PCR, the cycle threshold (C_t_) was determined for the housekeeping gene (TBP) as well as target genes using the auto baseline and auto threshold conditions. Normalized gene expression data, using ∆∆C_t_ (∆C_t_ reference- ∆C_t_ target) and the formula 2^-∆∆Ct^, were utilized for further analysis.

### Western blot

Western blot analysis was performed to analyze IDO expression and Akt/pAkt signaling pathway activation. Briefly, total protein was extracted from T24 cells by lysis buffer (50 mM Tris-Base, 1 mm EDTA, 0.5 μM PMSF, 0.001% Triton X-100, 0.1 mM AEBSF, 0.08 μM aprotinin, 4 μM bestatin, 1.4 μM E-64, 2.0 μM leupeptin and 1.5 μM pepstatin). One hundred micrograms of total protein were denatured and loaded on a 12% sodium dodecyl sulfate (SDS)-polyacrylamide electrophoresis gel and then transferred to a nitrocellulose membrane by semi-dry electroblotting (Trans-Blot SD Semi-Dry Transfer Cell; Bio-Rad Laboratories, Hercules, CA). After blocking with non-fat milk, the membranes were incubated with 1:250 mouse anti-human IDO (MAB5412; Merck Millipore, Billerica, MA) or 1:1000 rabbit anti-human phospho-Akt (MAB 2965; Cell Signaling Technology Inc., Danvers, MA). After detection by enhanced chemiluminescence (ECL-detection system; Amersham Pharmacia Biotech Inc., Piscataway, NJ), the anti-IDO and anti-phospho-Akt membranes were stripped with stripping buffer (62.5 mM Tris-HCl, 2% SDS, 0,1 M 2-mercaptoethanol), and incubated with 1:5000 mouse anti-human β-actin (A1978; Sigma-Aldrich, St. Louis, MO) and 1:1000 rabbit anti-human Akt (MAB 4685; Cell Signaling Technology Inc., Danvers, MA), respectively. For detection, 1:5000 goat anti-rabbit IgG HRP-conjugated (Sigma-Aldrich, St. Louis, MO) and 1:5000 goat anti-mouse IgG HRP-conjugated (Sigma-Aldrich, St. Louis, MO) secondary antibodies were used.

### Kynurenine measurement

HPLC was performed to measure kynurenine in the cell culture supernatants. The samples were deproteinized by centrifugation at 5,000 g (15 min at 4°C) with 10% trichloroacetic acid (1:1, v/v). In parallel, a standard curve was constructed with the following concentrations: 0.5 μM, 1.0 μM, 2.0 μM, 4.0 μM, 8.0 μM, and 16.0 μM. After centrifugation, the supernatants and the standards were filtered through a 0.22 μm syringe-loaded filter and resolved with a mobile phase of acetonitrile plus sodium acetate buffer (4:96, v/v), pH 4.7. A precolumn of 12.5X4.6 mm and a column of 250X4.6 mm (5 μm; Agilent Hypesil ODS, Agilent Technologies, Santa Clara, CA, USA) were used. The peak representing kynurenine was detected with the Chemstation Agilent software (G2170AA, Agilent Technologies, Santa Clara, USA).

### Scratch-wound migration and transwell invasion assays

For scratch-wound assays, T24 cells were seeded in 24-well plates (5X10^4^ per well) and cultured until reaching 80% confluence (24–30 h). Cells were maintained in serum-free McCoy’s 5A Medium for 24 h and then treated with the following (triplicate): MT (1 mM), TGF-β1 (5 ng/ml), and TGF-β1 + MT. Cells maintained in serum-free medium represented the control. After a 24 h incubation with the treatment, supernatants were removed and fresh medium was added (10% FBS). One scratch per well was carried out using a 200 μl pipette tip and four images per well were taken at 40X magnification under an inverted microscope (Ti-S; Nikon Corp., Tokyo, Japan). After three hours, additional images were acquired. Each scratch-wound area was calculated using the ImageProPlus 6.0 program (Media Cybernetics Inc., Bethesda, MD).

Transwell invasion assays were also performed. Transwells were fitted with membranes (6.5 mm diameter, 8.0 μm pore size, Corning Inc., NY), coated with BD Matrigel Basement Membrane Matrix (BD Biosciences, San Jose, CA) at a concentration of 3 mg/ml, and then incubated at 37°C for 2 hours. Cells were treated as described for the scratch-wound assay, and then seeded on top of the Matrigel coating with serum-free medium (triplicate). The cells were allowed to migrate for 6 h and 24 h towards a chamber containing McCoy’s medium supplemented with 20% fetal bovine serum. After incubation, the membranes were fixed in methanol and stained with hematoxylin. The number of migrated cells was determined under microscope magnification (400X).

### Animal model for invasion analysis

All animal procedures were approved by the Nove de Julho University Institutional Animal Care and Use Committee.

Three male BALB/c nude mice (5–6 weeks old) were used. The mice were maintained in individually ventilated cages under filtered air (Alesco, Capivari, Sao Paulo, Brazil) with free access to water and food. Intraperitoneal injection of ketamine (100 mg/Kg; Ketamin-S, Sao Paulo, Brazil) and xylazine (10 mg/Kg; Rompun, Bayer, Leverkusen, Germany) was utilized to achieve general anesthesia. A 10 mm lumbar incision was performed to access the left kidney. T24 cells (1X10^6^) were resuspended in 10 microliters of PBS and inoculated under the kidney capsule using a 21G-needle attached to a 100 μl-Hamilton syringe (Hamilton Company, Reno, Nevada). After careful inoculation, the capsular incision was subsequently cauterized. Mice were monitored daily for 28 days. On the 28^th^ day, mice were anesthetized with ketamine/xylazine, followed by laparotomy and thoracotomy to verify metastasis. For each mouse, a midcoronal section of the left kidney was fixed in Duboscq-Brazil solution for 60 minutes followed by post-fixation in buffered 10% formaldehyde solution until paraffin embedding. Sections (3 μm thick) were stained with hematoxylin and eosin and then used for immunohistochemistry. Histological analysis was used to verify the presence of T24 cells in the subcapsular space and renal parenchyma.

### Immunohistochemistry

Paraffin sections were subjected to microwave irradiation in citrate buffer to enhance antigen retrieval. Biotin Blocking System (X0590, Dako Co, Denmark) and non-fat milk blocking were used before primary antibody incubation. Mouse anti-human IDO (MAB5412; Merck Millipore, Billerica, MA) (1:25) was used as the primary antibody for the analysis of IDO expression. To complete the sandwich, sections were incubated with LSAB+ System-HRP reagents (K0690; Dako Co, Denmark). Finally, DAB substrate-chromogen was used to complete the reaction (K346811; Dako Co, Denmark).

### Statistical Analysis

Data are presented as the mean ± SEM. For parametric data, one-way analysis of variance with pairwise comparisons was conducted according to the Newman-Keuls formulation. For non-parametric data, Kruskal-Wallis or Wilcoxon was applied. A p-value less than 0.05 was considered significant.

## Results

### TGF-β1 and MT inhibit IDO expression

**Fig 1:**
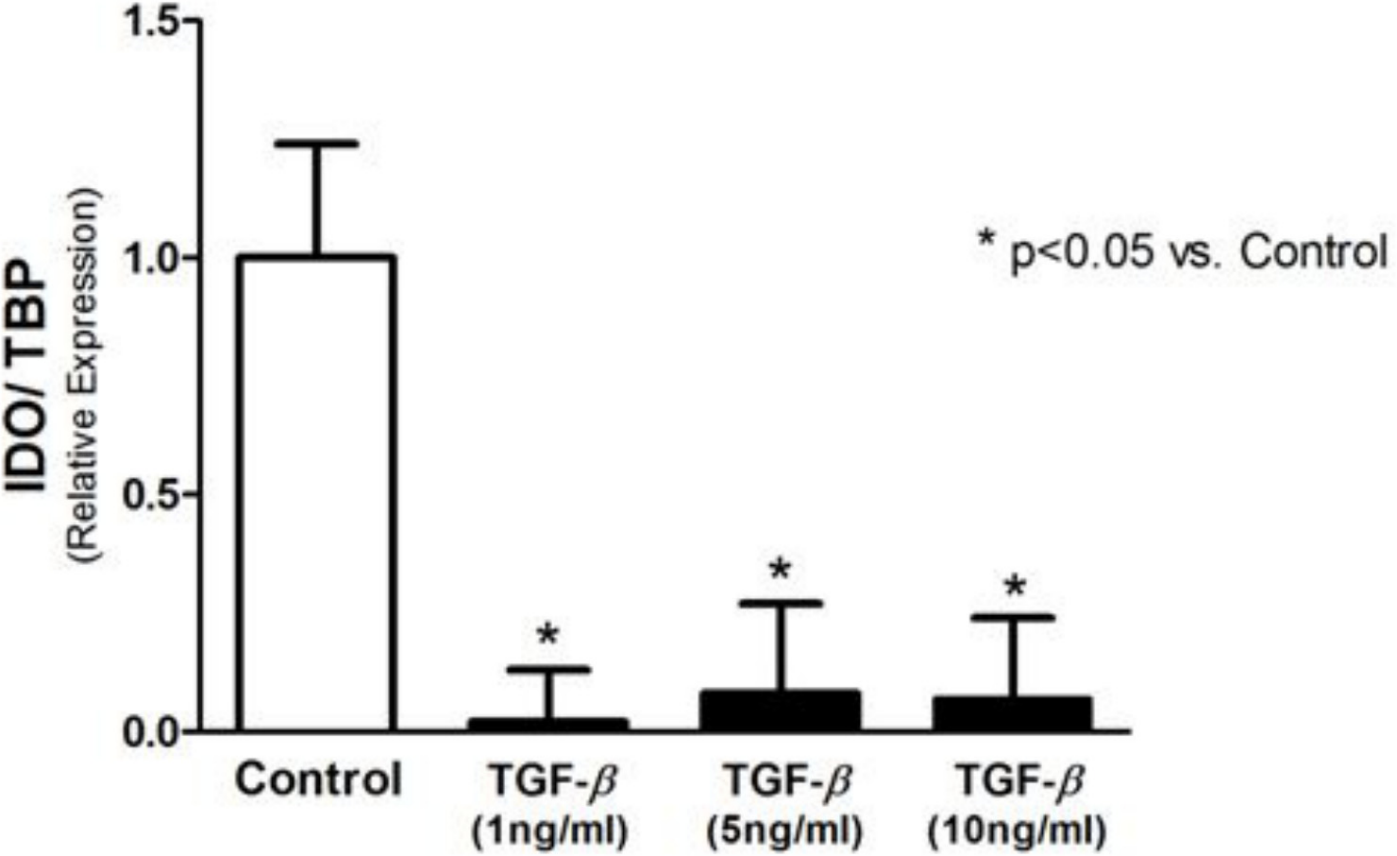
IDO expression analyzed by real-time PCR. IDO expression is downregulated by TGF-β in T24 cells.

**Fig 2:**
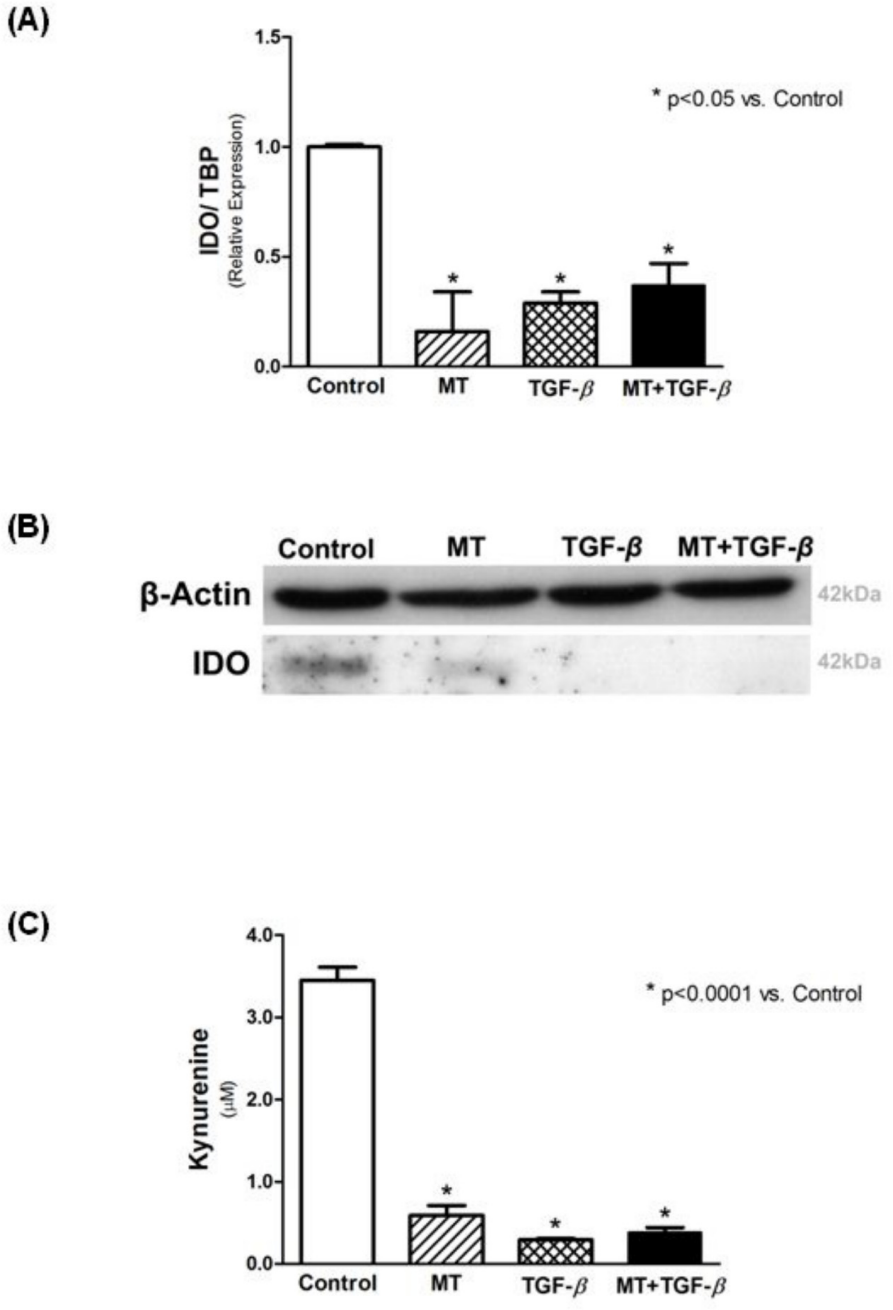
MT and TGF-β1 treatment diminishes IDO expression and activity in T24 cells. (A) IDO expression by real-time PCR, (B) Western blotting for IDO, and (C) IDO activity assessed by kynurenine measurement in the supernatant.

Incubation of T24 cells with TGF-β1 significantly decreased IDO expression (Fig 1), for all tested concentrations. As illustrated in Fig 2A, treatment with MT and MT + TGF-β1 significantly reduced expression of IDO in T24 cells (relative expression of 0.16 ± 0.18 vs. 1.00 ± 0.01). This effect was also observed in the results of Western blotting for the IDO protein (Fig 2B). To assess IDO activity, kynurenine was measured in the supernatant of the T24 cells using HPLC. The concentration of kynurenine was markedly diminished after incubation with MT, TGF-β1, and MT + TGF-β1 (0.59 ± 0.12 μM, 0.29 ± 0.02 μM, and 0.38 ± 0.06 μM, respectively, vs. 3.45 ± 0.16 μM in the Control; p<0.0001) (Fig 2C).

### MT potentiates EMT gene expression

**Fig 3:**
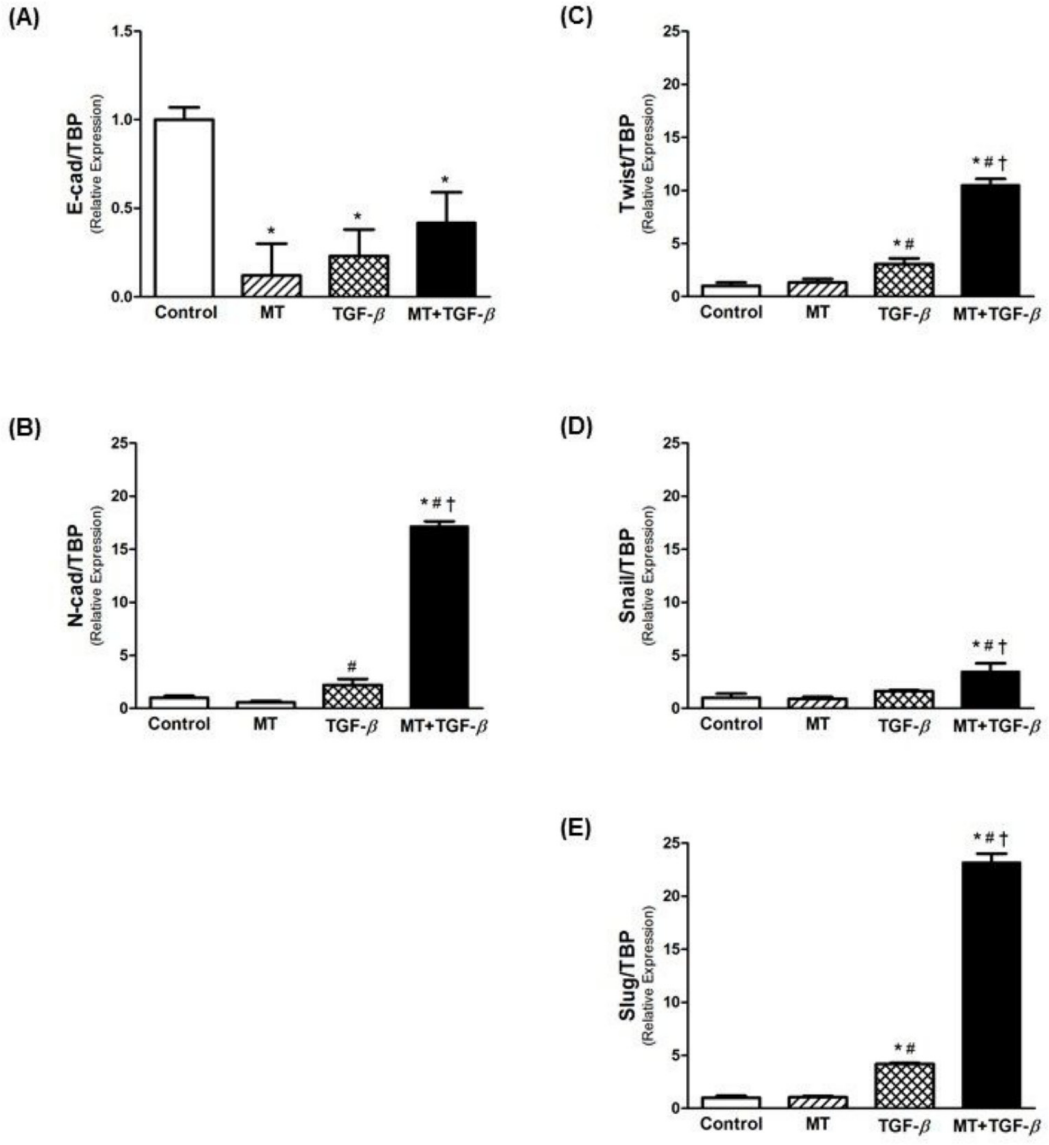
Real-time PCR for EMT markers. Relative mRNA levels of (A) E-cadherin (epithelial marker), (B) N-cadherin (mesenchymal marker), and (C-E) EMT-associated transcriptional factors (Twist, Snail and Slug). Association of MT and TGF-β1 potentiated EMT in T24 cells, decreasing E-cadherin expression and upregulating inductor genes of EMT. * p<0.05 vs. Control; ^#^ p<0.05 vs. MT; ^†^ p<0.05 vs. TGF-β.

A significant decrease was observed in Ecad expression with MT and TGF-β1 treatments (Fig 3A). In contrast, Ncad expression significantly increased with TGF-β1 treatment (Fig 3B), an effect that was potentiated by the combination of TGF-β1 with MT (more than 8 fold vs. TGF-β1 alone). In addition, the expression of EMT-associated transcription factors (Twist, Snail and Slug) was increased by TGF-β1, and treatment with TGF-β1 in combination with MT intensified this effect (Fig 3C, 3D and 3E).

### siRNA-mediated knockdown of IDO and expression of EMT markers

**Fig 4:**
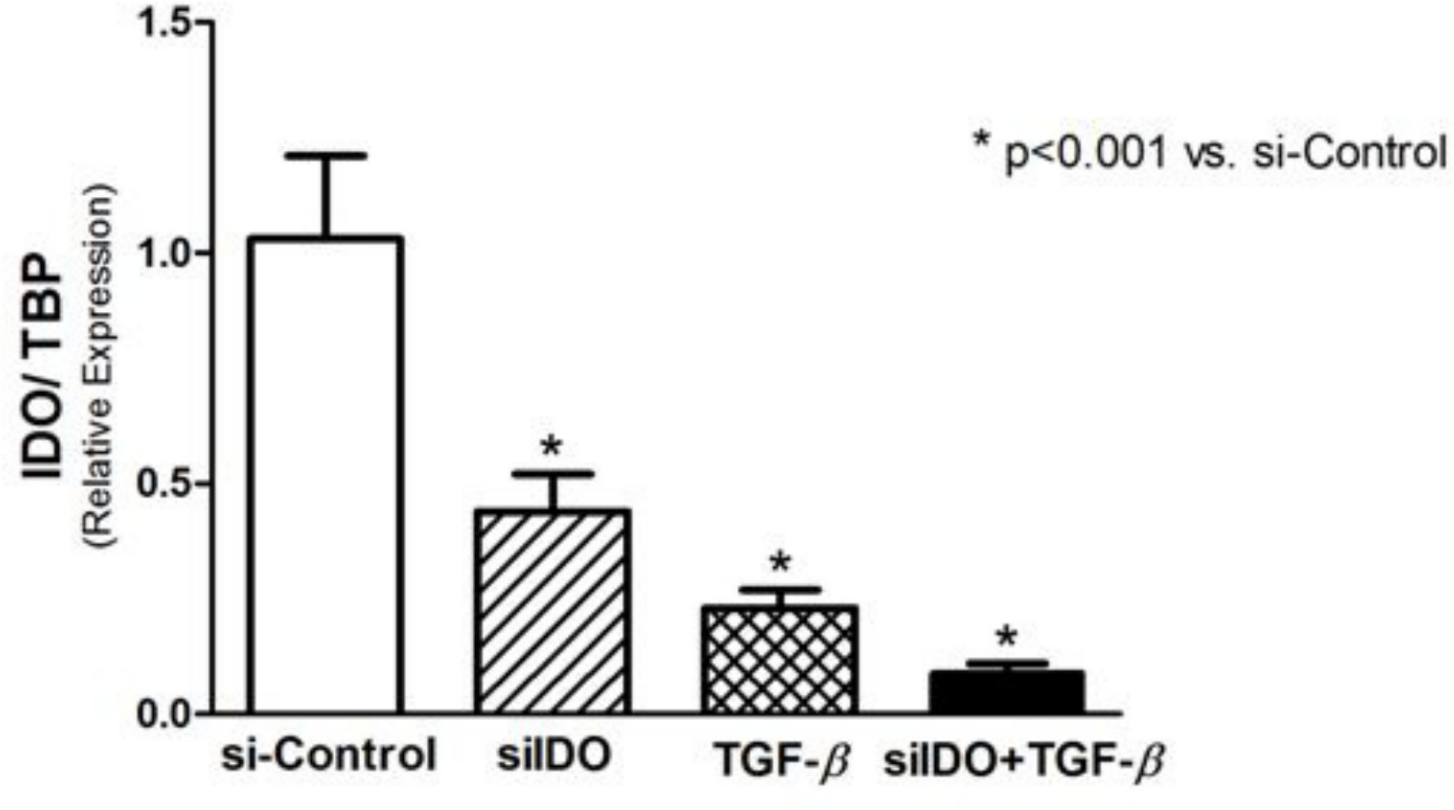
Real-time PCR for IDO to prove the effectiveness of the IDO-siRNA to induce IDO knockdown. A negative control-siRNA was used for si-Control and TGF-β conditions. A significant reduction of IDO expression was observed in siIDO cells, and in TGF-β-stimulated cells. The association of siIDO and TGF-β induced a more pronounced reduction of the IDO expression.

**Fig 5:**
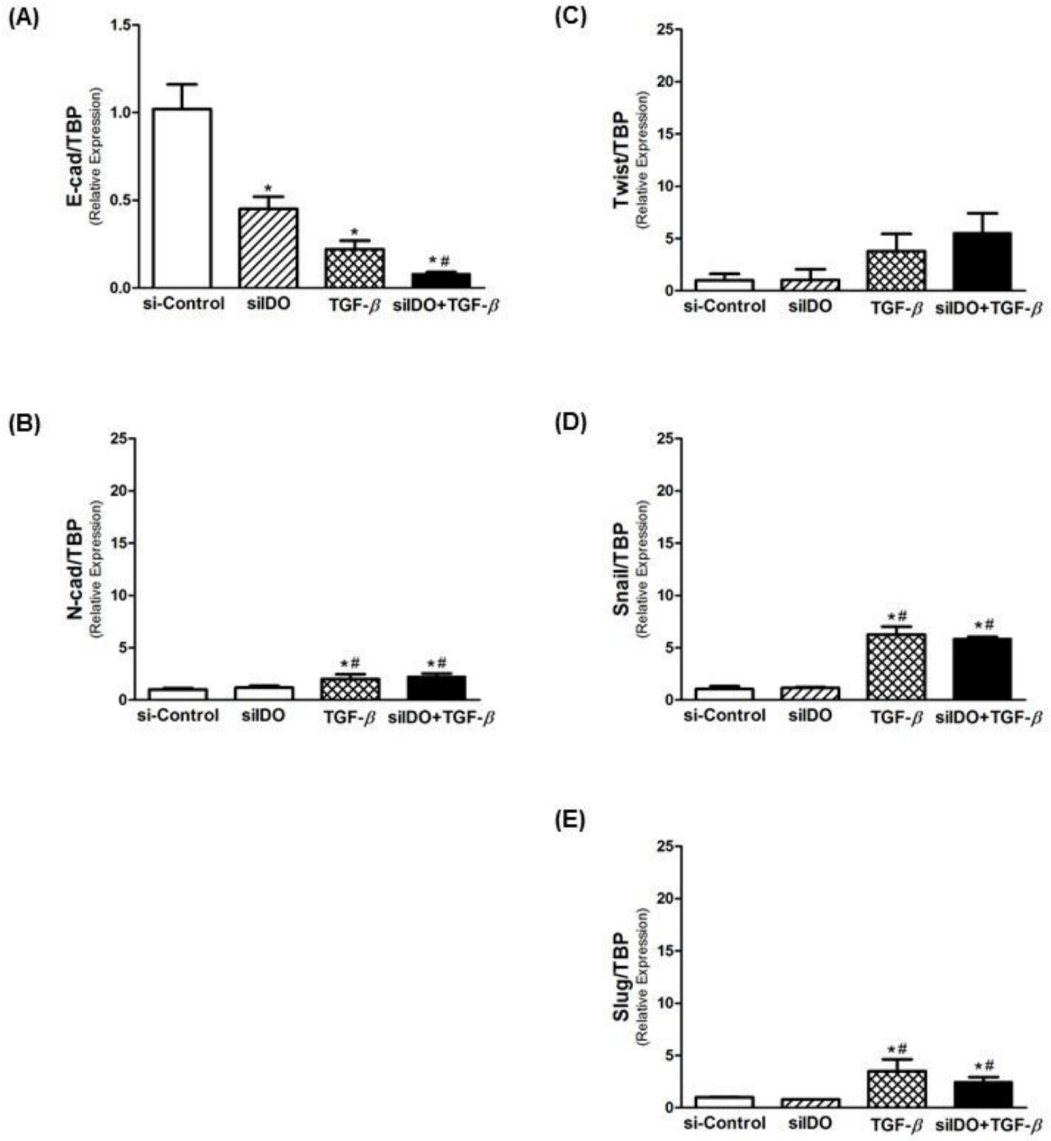
Effect of siRNA-mediated knockdown of IDO on EMT markers. Relative mRNA levels of (A) E-cadherin (epithelial marker), (B) N-cadherin (mesenchymal marker), and (C-E) EMT-associated transcriptional factors (Twist, Snail and Slug). siRNA-mediated knockdown of IDO was effective in reducing E-cad expression, and its association with TGF-β potentiated this effect. However, knockdown of IDO alone had no effect in N-cad expression, as well as in the expression of EMT-transcription factors. Only TGF-β acted inducing the expression of N-cad and EMT-transcription factors, and its association with siIDO did not change this effect. * p<0.05 vs. Control; ^#^ p<0.05 vs. MT.

The use of siRNA was effectiveness in silencing IDO gene expression. As demonstrated in Fig 4, siIDO significantly decreased the expression of IDO in T24 cells. A significant reduction of IDO expression was observed after stimulus with TGF-β1, effect that was intensified when associated with siIDO (Fig 4). Regarding the EMT markers, siIDO significantly reduced Ecad expression, and its association with TGF-β1 potentiates this effect, however, no statistical significance was found (Fig 5A). Although the siIDO showed effect on Ecad expression, siIDO had no effect in the expression of N-cadherin and EMT transcription factors (Twist, Snail, and Slug) (Fig 5).

### Scratch-wound and transwell invasion assays

**Fig 6:**
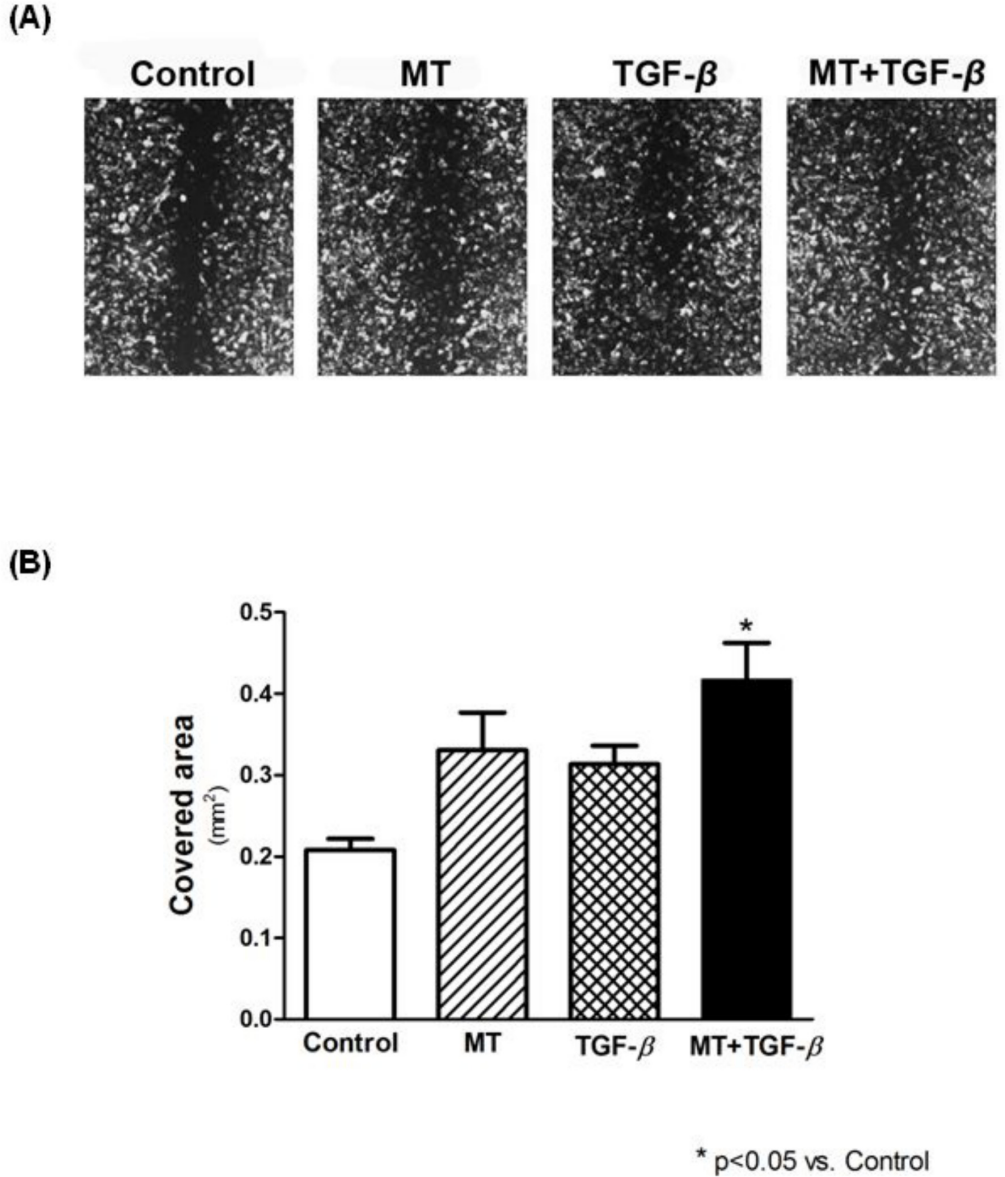
The scratch-wound assay was used to assess migration of T24 cells under MT and TGF-β1 stimulus. (A) Images were captured at 40X magnification under an inverted microscope, three hours after scratch formation. (B) Wound closure was significantly faster in MT + TGF-β compared to Control.

In the scratch-wound assay, the migration capacity of the T24 cells was evaluated by measuring the area occupied by migrating cells. As demonstrated in Fig 6, 3 hours after introduction of the scratch-wound, MT and TGF-β1 induced a faster migration of T24 cells compared to untreated cells. This heightened migration process was intensified when the cells were incubated with TGF-β1 plus MT (Fig 6A and 6B).

**Fig 7:**
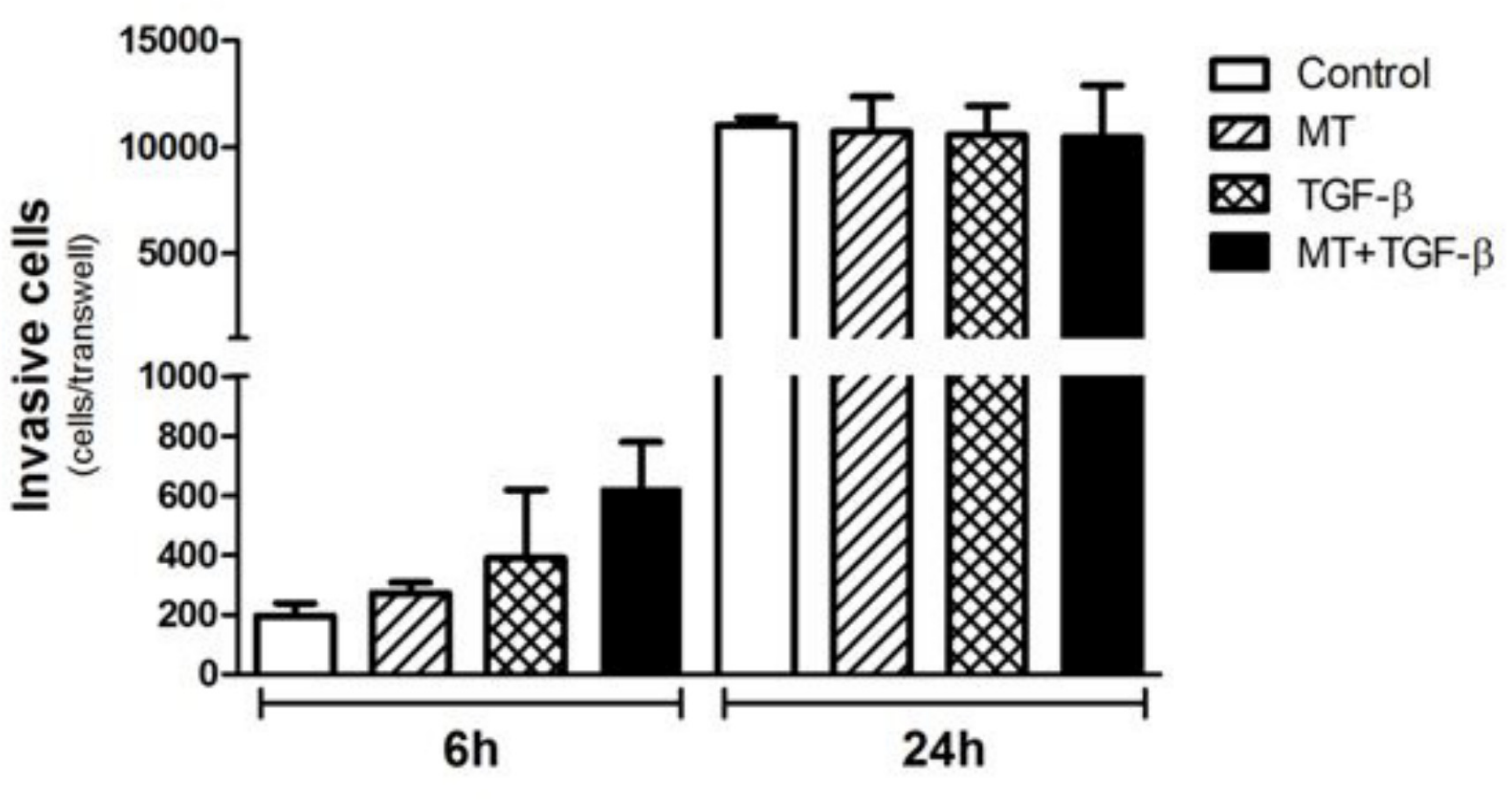
Analysis of the invasive potential of T24 cells by the Matrigel invasion assay. Although a greater level of invasive capacity was observed in T24 cells after 6 hours of TGF-β and MT + TGF-β treatment, no statistical significance was observed. After a migration period of 24 hours, no difference was found.

T24 cell invasion was studied using reconstituted Matrigel in transwell chambers. After 6 h of incubation, heightened invasion activity was observed when the cells were incubated with TGF-β1 plus MT, although the result was not statistically significant (Fig 7). After 24 h of incubation, no observable difference was detected between the different groups (Fig 7).

### Akt activation

**Fig 8:**
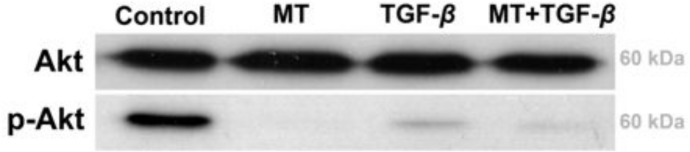
PI3K/Akt pathway analysis. PI3K/Akt pathway activation was assessed by Western blotting for Akt and phospho-Akt in T24 cell lysates. After 48 hours of incubation with MT, TGF-β, and MT + TGF-β, a decreased level of phospho-Akt was observed. Inhibition of the PI3K/Akt pathway is associated with EMT in T24 cells.

To analyze Akt pathway activation, Western blotting experiments were performed. As demonstrated in Fig 8, incubation of T24 cells with MT, TGF-β1 or MT + TGF-β1 promoted robust inhibition of Akt activation.

### *In vivo* analysis

**Fig 9:**
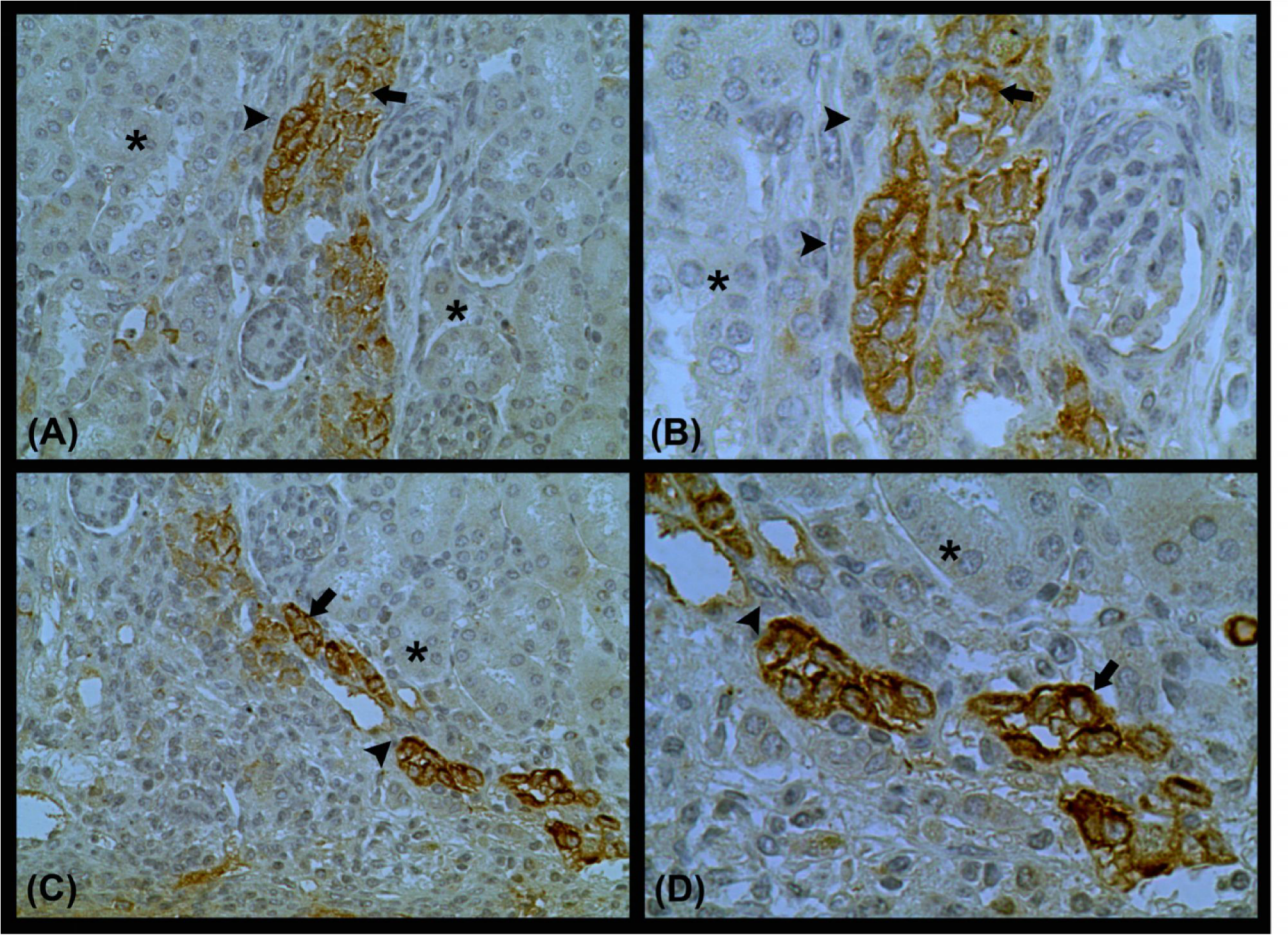
Immunohistochemistry for IDO expression in renal tissue 28 days after T24 cell inoculation. IDO-positive cells were found in the infiltrate center (arrow), while IDO-negative cells were predominantly found in the periphery of the infiltrate (arrowhead). IDO immunostaining was not observed in the renal parenchyma (asterisk).

To analyze IDO expression *in vivo*, T24 cells were inoculated under the kidney capsule of nude mice. Four weeks after the inoculation, we identified T24 cells under the kidney capsule. Furthermore, T24 cells were found infiltrating the renal parenchyma toward the renal medulla (S1 Fig). Immunohistochemical analysis of IDO expression revealed that subcapsular and infiltrating T24 cells were positive for IDO. However, the expression of IDO was specifically localized to the center of the cell infiltrate (Fig 9).

## Discussion

In the present study, we investigated the non-immunological role of MT and IDO in cancer progression and dissemination, particularly in EMT. Bladder carcinoma T24 cells constitutively express IDO, even in the absence of an immune system. We were able to induce EMT in T24 cells with TGF-β1, and this effect correlates with downregulation of IDO. When we use MT or IDO siRNA, the EMT was potentiated, especially by MT.

EMT is a process in which epithelial cells acquire an invasive phenotype, leading to a systemic spread of the malignant cells. During EMT, specific genes are expressed that ultimately function to deregulate intracellular biochemical pathways, which contributes to the establishment of a malignant phenotype. In our study, we assessed the process of decreased expression of E-cadherin followed by increased N-cadherin expression, a process called “cadherin switching,” to characterize EMT in T24 cells [29]. EMT plays an important role in bladder cancer, where motility, invasion, and anti-apoptotic mechanisms are required for metastasis [30].

TGF-β1 is a potent inducer of EMT in certain types of tissue, including bladder cancer. T24 cells express TGF-β receptor 1, and siRNA-based inhibition of TGF-β receptor 1 suppresses the motility and invasiveness of these cells [27]. In our study, T24 cells acquired mesenchymal characteristics, such as mesenchymal marker expression and motility, after TGF-β1 stimulus. Interestingly, TGF-β1 treatment induced EMT and inhibited IDO expression in T24 cells. In addition, MT, which also diminished IDO expression, intensified the TGF-β-driven EMT.

The siRNA-mediated knockdown of IDO significantly reduced the E-cadherin expression, but the mesenchymal markers were not altered. Because MT significantly increased the expression of mesenchymal markers, these results indicate that IDO pathway possibly participates in the EMT of T24 cells, but MT has an effect potentiating TGF-β1-induced EMT independently of IDO. Metz et al demonstrated that MT has effect on dendritic cells independently of IDO inducing the mTOR pathway (Metz). It is important to note that mTOR is strongly associated with EMT in bladder cancer [31]. It is possible that MT mediates mTOR pathway in T24 cells, but this mechanism was not assessed in our study.

Our study is the first to examine the relationship between TGF-β1 and IDO in T24 cells. However, there are studies demonstrating that TGF-β1 may modulate IDO expression in certain types of cells. Yuan et al. demonstrated that while IDO expression can be induced in human fibroblasts after stimulation with INF-gamma, TGF-β1 abrogates this effect without affecting INF-gamma expression [32]. In contrast, TGF-β1 induced IDO expression in dendritic cells via the PI3K/Akt and noncanonical NFK-β pathways, leading to a tolerogenic phenotype [26]. The mechanisms linking TGF-β and IDO have not been completely elucidated, but a more plausible mechanism has been described by Pallotta and Grohmann [33]. Their studies support the theory that TGF-β confers regulatory effects on IDO synthesis. Furthermore, IDO may exert its effects through a mechanism that is independent of its enzyme activity [33].

In our study, we assessed PI3K/Akt pathway activation using Western blotting for Akt/phospho-Akt. Treatment with MT and TGF-β1 significantly decreased phospho-Akt levels, indicating that TGF-β-driven EMT in T24 cells is associated with the downregulation of this pathway. In many cell types, TGF-β-induced EMT is associated with PI3K/Akt pathway upregulation; however, in T24 cells it remains unclear. Al-Azayzih et al. analyzed phospho-Akt in T24 cells after incubation with TGF-β1 (as used in our experiments, 5 ng/ml), and they were unable to determine a difference in phospho-Akt levels [34]. The absence of effect on phospho-Akt levels may be due to the length of TGF-β1 treatment. While they used an incubation period of 24 hours, we used 48 hours. Furthermore, Iliopoulos et al. used Akt-/- cells to show that Akt1 knockdown promoted TGF-β-induced EMT in MCF10A cells. This effect was shown to be dependent on the expression of specific microRNAs [35]. In this context, the role of Akt in EMT may differ depending on the model system.

In addition to cadherin switching and significantly increased expression of EMT-related transcription factors (Twist, Snail, and Slug), T24 cells treated with TGF-β plus MT demonstrated increased migratory capacity and invasiveness. In addition to *in vitro* experiments, T24 cells were inoculated in the kidney subcapsular space of Balb/c nude mice. After 4 weeks, T24 cells were observed in the renal parenchyma toward the medulla. Immunohistochemical analysis demonstrated that T24 cells in the center of the infiltrate were positive for IDO, while peripheral cells were IDO-negative. It is possible that the peripheral cells of the infiltrate lost IDO expression to acquire an invasive phenotype. Further studies are necessary to clarify this mechanism.

In conclusion, MT and downregulation of IDO are associated with EMT in T24 human bladder carcinoma cells, and this effect may be triggered by TGF-β1. Although IDO inhibition is attractive as anti-cancer therapy because IDO promotes tolerance to tumors, the nonimmunological effects mediated by MT and other IDO modulators deserve consideration in bladder cancer. As perspective, inhibitors of IDO must be combined with other classes of drugs in anti-cancer therapy to minimize possible adverse effects caused by these agents.

## Supporting Information

S1 Fig Histology of the renal tissue after subcapsular inoculation of T24 cells. To analyze T24 cell invasion *in vivo*, we inoculated 1X10^6^ cells under the kidney capsule. Histology (hematoxylin and eosin staining) was assessed 28 days after inoculation. (A) T24 cells under the kidney capsule (arrowhead), and (B) T24 cells infiltrating the renal parenchyma toward the renal medulla (arrow). Renal parenchyma is indicated with asterisk.
(TIF)
