# Supplementary figures and images for: 1-Methyl-D-Tryptophan Potentiates TGF-β-Induced Epithelial-Mesenchymal Transition in T24 Human Bladder Cancer Cells
Source: PLoS One. 2015 Aug 12;10(8):e0134858. doi: 10.1371/journal.pone.0134858 (PMC4534444; doi:10.1371/journal.pone.0134858)

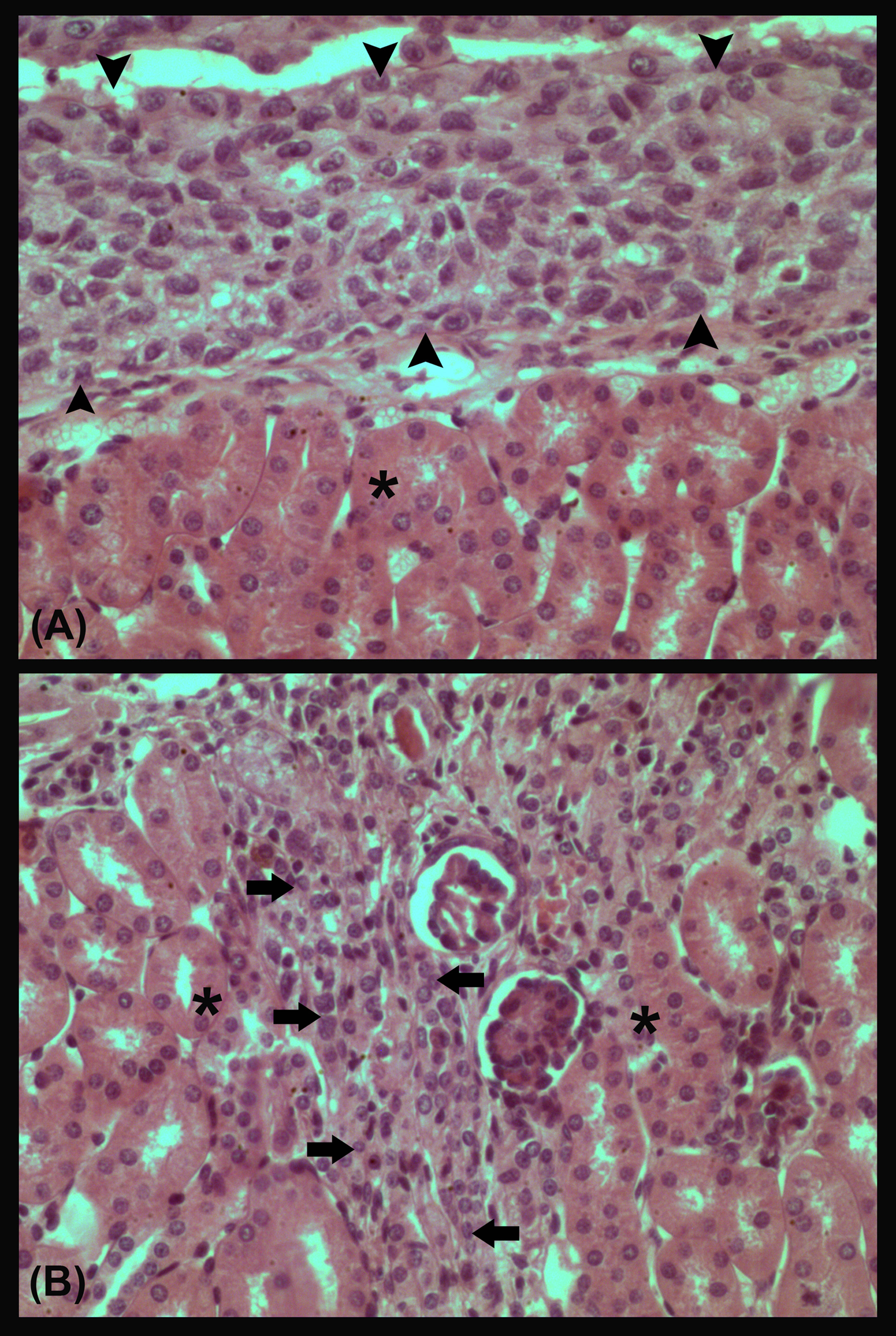

Supplement: S1 Fig — To analyze T24 cell invasion in vivo, we inoculated 1X106 cells under the kidney capsule. Histology (hematoxylin and eosin staining) was assessed 28 days after inoculation. (A) T24 cells under the kidney capsule (arrowhead), and (B) T24 cells infiltrating the renal parenchyma toward the renal medulla (arrow). Renal parenchyma is indicated with asterisk. (TIF) [file pone.0134858.s001.tif]
